# Characterization of wheat (*Triticum aestivum*) *TIFY* family and role of *Triticum Durum Td*TIFY11a in salt stress tolerance

**DOI:** 10.1371/journal.pone.0200566

**Published:** 2018-07-18

**Authors:** Chantal Ebel, Asma BenFeki, Moez Hanin, Roberto Solano, Andrea Chini

**Affiliations:** 1 Plant Physiology and Functional Genomics Research Unit, Institute of Biotechnology, University of Sfax, BP Sfax, Tunisia; 2 Plant Molecular Genetics Department, Centro Nacional de Biotecnología, Consejo Superior de Investigaciones Científicas (CNB-CSIC), Madrid, Spain; Institute of Genetics and Developmental Biology Chinese Academy of Sciences, CHINA

## Abstract

The TIFY proteins constitute a plant-specific super-family and they are involved in regulating many plant processes, such as development, defences and stress responses. The Jasmonate-ZIM-Domain (JAZ) proteins, the best-characterized sub-group of the TIFY family are key regulator of the jasmonic acid (JA) signalling pathway. Jasmonates regulate several aspects of plant development, and play a primary role in defence mechanisms as well as in plant responses to abiotic stresses. The TIFY family is well studied in dicots but poorly investigated in monocots. The present study reports an extensive genomic identification of TIFY proteins from *Triticum aestivum*. We identified 49 *TIFY* genes, which were annotated according to three sub-genomes (AABBDD) of *T*. *aestivum*. Following their clustering with *Oryza sativa* and *Brachypodium distachyon*, the 49 genes were grouped in 18 different *TIFY* homeologous subsets. Expression analyses of 6 representative *TIFY* genes on Tunisian durum wheat seedlings revealed their differential regulation by various stress treatment, including JA, ABA and salt stress. *TIFY11a* was specifically induced after salt treatment. Transgenic lines over-expressing *Td*TIFY11a showed higher germination and growth rates under high salinity conditions, compared to wild type plants. In summary, our results outline a relevant role of wheat TIFY proteins in promoting germination under salt stress.

## Introduction

Because of their sessile lifestyle, plants have evolved myriads of defense mechanisms to survive the continuous challenges of their ever-changing environment, including exposure to pathogens and insects but also, droughts, salty soils or mineral deficiency. Many signaling pathways participate in plant adaptation to environmental cues. Plant hormones are major actors of plant defense against environmental changes and among them abscisic acid (ABA) is considered as the abiotic stress hormone while jasmonic acid (JA) is traditionally regarded as the hormone that regulates plant defenses to necrotrophic pathogens, fungi, insect and nematodes[1–3].

The basic signaling mechanisms orchestrating JA-responses have been deciphered[2,4]. In response to stresses or endogenous signals, plants accumulate the active form of the hormone, (+)-7-iso-JA-Ile (JA-Ile), the ligand of the co-receptor complex formed by the F-box protein coronatine-insensitive 1 (COI1) and the co-receptor Jasmonate ZIM-domain (JAZ)[5,6]. The JA-Ile-mediated COI1-JAZ interaction promotes the ubiquitination and degradation of the JAZ repressors that liberates several transcription factors (TFs) including the JA master regulator MYC2[7–9], a basic helix-loop-helix (bHLH) DNA-binding protein. In turn, these TFs trigger JA-specific cellular outputs such as defense responses or inhibition of plant growth[2,4]. The conserved Jas domain of the JAZ repressors mediates the interaction with MYC2, but also with other TFs of different families such as other bHLHs, MYBs, YABBYs, WRKY, AP2 and EIN3/EIL3[1,2,4]. In addition, alternative splicing, resulting in the retention of the Jas-intron, encodes truncated JAZ variants that act as constitutive repressors of the JA-pathway, such as the case of the *Arabidopsis JAZ10.4[10–12]*. Additional truncated JAZ variants lacking the Jas motif also confer dominant insensitivity to JA[4,10,12].

All JAZ proteins retain the conserved ZIM (Zinc-finger expressed in Inflorescence Meristems) or TIFY domain, and therefore they belong to the plant specific family called TIFY family that includes JAZ, TIFY8, ZIM-like (ZML) and PEAPOD (PPD) proteins which have been particularly well studied in *Arabidopsis*[13]. These *Arabidopsis* proteins all possess a conserved TIFY or ZIM domain composed of 36 amino acids containing a core motif TIF[F/Y]XG[13]. The TIFY domain is required for JAZ dimerization and mediates the interaction with NINJA (Novel Interactor of JAZ), which recruits the TOPLESS (TPL) general transcriptional co-repressor[11,14,15].

In addition to the TIFY domain, ZMLs possess a C2C2-GATA zinc-finger DNA-binding domain and a CCT-domain (CONSTANS, CO-like, TOC1) that is closely related to the Jas domain in JAZ proteins[16]. In contrast, the PPD proteins, beside the TIFY domain, harbor at their N-terminus a typical PPD domain[13,17]. Some TIFY proteins, such as AtJAZ7 and AtJAZ8, hold an EAR motif (ethylene-responsive element binding factor-associated amphiphilic repression) that enables them to directly recruit the TPL co-repressor[18].

Beyond *Arabidopsis*, TIFY families have been recently described in several plant species, including tomato, rice, maize and *Brachypodium*[13,19–22]. Different functions have been described for TIFY proteins belonging to different subfamilies. For example, TIFY8, PPD and ZML proteins are involved in the transcriptional regulation of developmental processes. In *Arabidopsis*, loss-of-function mutations of PPD1 and PPD2 affect leaf shape, silique length modifications and meristemoid division[17,23], while the leguminous ortholog PPD gene *BIG SEEDS1* regulates cell proliferation and plant organ size[24]. AtTIFY1/ZML over-expression results in hypocotyl elongation while ZML2 acts as a transcriptional repressor in lignin biosynthesis in maize[16,25]. In all plant species studied, JAZ proteins are the most represented groups in TIFY families. *Arabidopsis* possess 13 different JAZ members with extensive redundancy, but also specific functions[4]. For instance, AtJAZ12 is specifically degraded after interaction with the ABA repressor-E3 Ubiquitin ligase KEG (KEEP on GOING)[26]. *AtJAZ2* is expressed only in stomata where it triggers stomatal closure to hinder pathogen penetration[27].

JAZ proteins are also involved in abiotic stress tolerance mechanisms[19–21,28–30]. Enhanced stress tolerance of transgenic lines over-expressing JAZ proteins have been described in rice, cotton and wild soybean[20,31–33]. For example, rice lines overexpressing OsJAZ9/OsTIFY11a are salt and drought tolerant compared to WT plants[20,31].

Wheat is one of the most consumed cereals worldwide and its production is highly sensitive to environmental constraints[34]. Modulation of the JA pathway could be a novel strategy for biotechnological improvement of its productivity. However, little is known about the wheat TIFY proteins. Recently, 14 homeologous *JAZ* genes have been identified in *Triticum aestivum* L.[29] but a complete view of the wheat TIFY family is still lacking. Here, we provide a complete identification and characterization of *Triticum aestivum* TIFY protein family and the first evidence that the wheat *JAZ/TIFY* genes are involved in plant salt stress tolerance.

## Materials and methods

### Plant material and stress treatments

Seeds of Tunisian durum wheat variety Oum Rabiaa3 provided from INRAT (Tunisian Agronomic Research Institute) were surface sterilized with 1.5% (v/v) sodium hypochlorite for 15 min with gentle agitation, rinsed three times with sterile water and grown on wet Whatman paper, for 2 days in the dark, and for a week in a growth chamber at 23°C, under a 16 h photoperiod (16 h light/8 h dark) and 60% relative humidity. Stress treatments were done on ten 7-day-old seedlings using 150 mM NaCl, 50 μM JA, 100 μM ABA for 1 and 6 h.

*Arabidopsis* Col-0 seeds were obtained from the NASC Stock Center and used for transformation using the floral dip method[35]. For salt tolerance tests, after seed surface-sterilization and vernalization for 2 days at 4°C, seeds were grown on MS medium (0.5x, 0.7% agar) supplemented or not with NaCl (100, 150 or 200 mM).

Germination rates of 20 to 50 seeds were evaluated by observation of radicle emergence and cotelydon greening at 2 and 5 days after germination (DAG) respectively. Similar results were obtained in at least 4 independent biological replicates.

Root growth inhibition and accumulation of anthocyanins of 10-to-30 10-day-old seedlings grown in absence or presence of 50 μM JA were analyzed as described in[36].

### Identification of *Triticum aestivum TIFY* gene family and phylogenetic analyses

Common wheat TIFY protein sequences were retrieved by combining HMMER, BLAST analyses using *Oryza sativa* and *Brachypodium distachyon* TIFY proteins[20,21] as query on TGACv1 genome from EnsemblPlant (http://plants.ensembl.org/Triticum_aestivum/Info/Index) and phytozome databases (https://phytozome.jgi.doe.gov) as well as keyword searches using TIFY and JAZ as queries. The retrieved proteins have been analyzed using Pfam (http://pfam.xfam.org/) to ensure the presence of the TIFY domain.

The wheat TIFY proteins were aligned using MEGA 6.06 together with *Brachypodium distachyon* and *Oryza sativa* TIFY proteins[37]. Based on multiple alignment (CLUSTALW, Blosum matrix with default settings), pairwise comparison and phylogenetic analyses, we assigned to the 49 wheat different proteins their TIFY name. The phylogenetic tree was constructed using MEGA6.06 and the Neighbor-end joining method based on the number of aa substitutions.

### RNA extraction and gene expression analyses

Wheat total RNA extraction was performed on aerial parts of ten 7 day-old seedlings of durum wheat variety Oum Rabiaa3 treated as above-mentioned using Trizol reagent (Invitrogen) with manufacturer’s recommendations. The RNA was cleaned up from DNA contamination using on-column DNAse I removal kit (Roche). 1 μg of total RNA was used for reverse transcription using cDNA synthesis kit (Roche). After 1/10^th^ dilution, 5 ml of cDNA was used as a template for QPCR analyses in a total volume of 15 ml using Power SYBR Master mix (Applied Biosystems) as previously described[38]. Amplification and quantification was performed in a 7500 Real Time PCR system (Applied Biosystems). Wheat *Actin* gene (TRIAE_CS42_1AS_TGACv1_020044_AA0074210) was used as internal control. Quantification was performed using the ΔΔCt method[39] using actin and time 0 as references. Actin and TIFY primer pairs are reported in S1 Table.

A RNA isolation kit (FavorGen) was employed to extract Arabidopsis total RNA using biological samples of tissue pooled from 10–15 5-day-old seedlings. RNA was extracted including DNase digestion to remove genomic DNA contamination. cDNA was synthesized from 1.5 μg total RNA with the high-capacity cDNA reverse transcription kit (Applied Biosystems). For gene amplification, 4 μl from a 1:10 cDNA dilution was added to 4 μL of EvaGreen® qPCR Mix Plus (Solis BioDyne) and gene-specific primers previously described[38]. Quantitative PCR was performed in 384-well optical plates in a HT 7900 Real Time PCR system (Applied Biosystems) using standard thermo cycler conditions (an initial hold at 95°C for 10 min, followed by a two-step SYBRPCR program of 95°C for 15 s and 60°C for 60 s for 40 cycles). Relative expression values are the mean ± SD of three to four technical replicates relative to the basal wild-type control using ACT8 as housekeeping gene.

### *TdTIFY11a* isolation and cloning

Using cDNA sequences of *Triticum aestivum Ta*TIFY11a, primers were designed for PCR amplification of either the complete ORF or a truncated form lacking the Jas domain (ΔJas). For the full-length TIFY11a cloning, a first PCR amplification using JAZ2bisF1 (5’-CGGTTGGTGGAGTGCTTAGC-3’) and JAZ2bisR1 (5’-TGTACCAACGTTGCCGTGCA-3’) was done on wheat cDNA of Oum Rabiaa3 Tunisian durum wheat variety by adding 1% DMSO using the following program: 94°C, 30 s; 58°C, 30 s; 72°C 1 min. One microliter of this first 625bp-PCR product was used for nested PCR amplification using JAZ2bisF2 (5’-AAGGCCATCGATCGCCACCG-3’) and JAZ2bisR2 (5’-TGTTGAGGCGATCATTCACG-3’) and an annealing temperature of 58°C. A single 584 bp-band was observed and cloned into the pGEMTeasy vector (Promega) giving rise to the pTIFY11a-FL clone which was then confirmed by sequencing using the dye terminator cycle sequencing method (Applied Biosystems).

This clone was used for gateway cloning in the binary expression vector pEarleyGate 103, which was performed as follows. First, to attach the attB1/attB2 sites PCRs were carried out on pTIFY11a-FL clone using JAZ2bisF4B1 (5’-GGGGACAAGTTTGTACAAAAAAGCAGGCTTCATGCCGC-CGATGGCGACCA-3’) and JAZ2bisR4B2 (5’-GGGGACCACTTTGTACAAGAAAGCTGGGTCCGGCGCGTGCATGTCCCCTA-3’) for the full-length cDNA and JAZ2bisF4B1 (5’-GGGGACAAGTTTGTACAAAAAAGCAGGC-TTCATGCCGCCGATGGCGACCA-3’) and JAZ2bisΔjasR5B2 (5’-GGGGACCACTTTGTACAAGAAAGCTG-GGTCGACAAGCAAGGCTGCCCC-3’) for the truncated ΔJas version using the following programs respectively: 94°C, 30 s; 58°C, 30 s; 72°C 1 min. Second, the two distinct PCR products were cloned in pDONR207 in a BP reaction with BP clonase (Invitrogen). After sequencing, we performed a LR recombination step of the two clones with the binary vector pEarleyGate 103 that ensures an in frame C-terminal GFP fusion.

### Protein extraction and western blotting

A minimum of 20 mg of seedlings were collected and frozen in liquid nitrogen before quick grinding in sample buffer (0.5 M Tris-HCl (pH 8.5), 4% (w/v) lithium dodecyl sulfate, 20% (v/w) glycerol, 1 mM EDTA, 0.25 M DTT and tracking dye) to extract total proteins. The extraction was followed by 15 min centrifugation at 13 000 rpm and boiling at 100°C. The proteins were then separated on a 12% SDS-PAGE. After transfer on nitrocellulose membrane using the Mini-transfer system (BioRad) for one hour at 100 V, the blot was blocked during one hour in PBS, 5% milk. Then, the blot was incubated with anti-GFP antibody HRP conjugated (1/1000) for 1 hour. Detection was performed using the West Femto chemiluminescent signal detection kit (Pierce). Equal loading of total proteins was assessed by blotting the same membrane with mouse anti-actin antibody (1/2000) for 1 hour in PBS, 0.05% milk followed by incubation with anti-mouse IgG-HRP conjugated (1/10000; Roche). Detection was performed using the micro chemiluminescent signal detection kit (Pierce).

### Bioinformatic tools and statistical analyses

MEME suite (http://meme-suite.org/tools/meme) was used with default settings to identify conserved motifs within TIFY proteins. TIFY proteins were represented on scale using GPS1.0 drawing tool.

Statistical analyses were performed using One-way ANOVA with post-hoc Tukey HSD Test for comparing multiple treatments.

## Results

### Common wheat TIFY protein family

**Fig 1:**
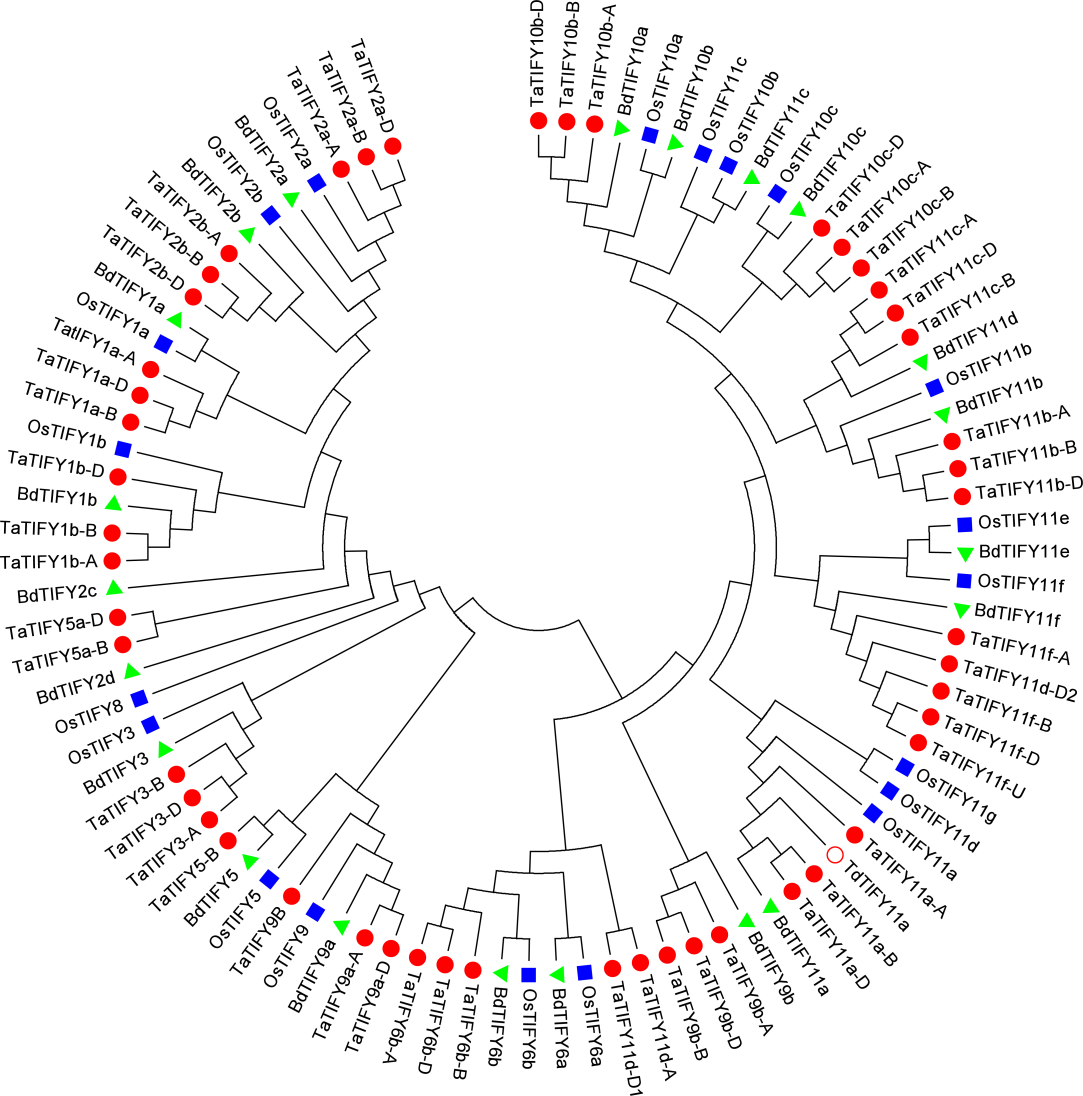
Phylogenetic tree of common wheat, Brachypodium and rice TIFY proteins. Phylogenetic tree was obtained using MEGA6.06 with the Neighbor-Joining method based on TIFY protein sequences. Wheat, Brachypodium and rice gene identifiers are indicated such as in Table 1. Wheat proteins are indicated by red dots, rice proteins by dark blue squares and Brachypodium proteins by green triangles. The red open circle represent durum wheat *Td*TIFY11a. Scale bar indicates evolutionary distances inferred using the Neighbor-Joining method calculated by the number of amino acid substitutions per site as conducted by MEGA6.06.

The different members of common wheat TIFY family proteins were retrieved by performing BLAST searches on Uniprot (http://www.uniprot.org) and Phytozome (https://phytozome.jgi.doe.gov/pz/portal.html) databases using available protein sequences of rice and Brachypodium TIFY proteins[20,21]. These searches allowed us to identify 49 *T*. *aestivum TIFY* genes, of which 15 were novel. Following their clustering with rice and *Brachypodium*, these 49 *TaTIFY* genes were grouped into 16 homeologous loci, with one gene copy on each of the three wheat subgenomes (*T*. *aestivum* AABBDD), and annotated accordingly (ie. -A; -B; -D) (Fig 1 and Table 1).

**Table 1:** List of the common wheat (*Triticum aestivum*) TIFY genes and proteins identified.

Gene Name	Locus ID	Uniprot Ref	TIFY motif	Protein length (aa)	Synonym Wang et al.2017
*TaTIFY1a-A*	TRIAE_CS42_4AL_TGACv1_292093_AA0997710	A0A1D5X965	TLLFQG	185	TaJAZ4-A
*TaTIFY1a-B*	Traes_4BS_8C20E76AA		TLLFQG	211	TaJAZ4-B
*TaTIFY1a-D*	Traes_4DS_AFCEDDE67		TLLFQG	315	TaJAZ4-D
*TaTIFY1b-A*	Traes_4AL_B6992AAA6		TLVYQG	270	TaJAZ5-A
*TaTIFY1b-B*	Traes_4BS_ACD70539F		TLVYQG	269	TaJAZ5-B
*TaTIFY1b-D*	TRIAE_CS42_4DS_TGACv1_361237_AA1164060	A0A1D5Y621	TLLYQG	261	TaJAZ5-D
*TaTIFY2a-A*	Traes_6AS_4106E8E28		TLSFQG	304	TaJAZ11-A
*TaTIFY2a-B*	TRIAE_CS42_6BS_TGACv1_513579_AA1645090	A0A1D6AWV1	TLSFQG	342	Not assigned
*TaTIFY2a-D*	TRIAE_CS42_6DS_TGACv1_543297_AA1738120	A0A1D6BEN9	TLSFQG	342	Not assigned
*TaTIFY2b-A*	Traes_7AL_EA6F4FFDE		TLSFQG	256	TaJAZ14-A
*TaTIFY2b-B*	Traes_7BL_7DC689032		TLSFQG	165	TaJAZ14-B
*TaTIFY2b-D*	Traes_7DL_A5ECEDA95		TLSFQG	247	TaJAZ14-D
*TaTIFY3-A*	Traes_2AL_46576945E		TIFYGG	152	Not assigned
*TaTIFY3-B*	Traes_2BL_0614A2B97		TIFYGG	236	TaJAZ2-B
*TaTIFY3-D*	Traes_2DL_64F090209		TIFYGG	188	Not assigned
*TaTIFY5-B*	TRIAE_CS42_2BL_TGACv1_129673_AA0392230				
*A0A1D5TVW8*	TIFYNG	159	Not assigned		
*TaTIFY5a-B*	TRIAE_CS42_7BL_TGACv1_576994_AA1862250	A0A1D6C372	TMTFRG	261	Not assigned
*TaTIFY5a-D*	TRIAE_CS42_7DL_TGACv1_605652_AA2007500	A0A1D6CUX5	TMTFRG	329	Not assigned
*TaTIFY6b-A*	Traes_5AL_BF3D7E764		TIFYAG	284	TaJAZ10-A
*TaTIFY6b-B*	Traes_5BL_1F38B9D05		TIFYAG	416	TaJAZ10-B
*TaTIFY6b-D*	Traes_5DL_3A1F8C38E		TIFYAG	418	TaJAZ10-D
*TaTIFY9-B*	Traes_2BL_E94651AAE		TVFYNG	133	Not assigned
*TaTIFY9a-A*	Traes_2AL_6CBE19B87		TVFYNG	170	TaJAZ3-A
*TaTIFY9a-D*	Traes_2DL_7DD4A39D4		TVFYNG	170	TaJAZ3-D
*TaTIFY9b-A*	Traes_6AL_BC7FB0A99		TIFYAG	267	TaJAZ12-A
*TaTIFY9b-B*	TRIAE_CS42_6BL_TGACv1_499397_AA1581290				
*A0A1D6AKJ0*	TIFYAG	321	Not assigned		
*TaTIFY9b-D*	Traes_6DL_ 7024F5429		TIFYAG	267	TaJAZ12-D
*TaTIFY10b-A*	Traes_2AS_A8CCC32D3		TIFYGG	231	TaJAZ1-A
*TaTIFY10b-B*	Traes_2BS_2C79AE2DE		TIFYGG	231	TaJAZ1-B
*TaTIFY10b-D*	Traes_2DS_C0C75D1D7		TIFYGG	231	TaJAZ1-D
*TaTIFY10c-A*	Traes_5AL_BB55F989A		TIFYGG	151	TaJAZ9-A
*TaTIFY10c-B*	Traes_5BL_7A6C3831E		TIFYGG	230	TAJAZ9-B
*TaTIFY10c-D*	Traes_5DL_4186C5347		TIFYGG	230	TaJAZ9-D
*TaTIFY11a-A*	TRIAE_CS42_4AS_TGACv1_307330_AA1019560				
*A0A1D5XCM2*	TIFYGG	175	Not assigned		
*TaTIFY11a-B*	TRIAE_CS42_4BL_TGACv1_320580_AA1043710	A0A1D5XHE6	TIFYGG	163	Not assigned
*TaTIFY11a-D*	TRIAE_CS42_4DL_TGACv1_343139_AA1130400				
*A0A1D5XZE1*	TIFYGG	163	Not assigned		
*TaTIFY11b-A*	Traes_4AS_6EAA11AAD		TIFYGG	137	TaJAZ6-A
*TaTIFY11b-B*	TRIAE_CS42_4BL_TGACv1_320580_AA1043690				
*A0A1D5XHD9*	TIFYGG	187	Not assigned		
*TaTIFY11b-D*	Traes_4DL_E25D3DF01		TIFYGG	181	TaJAZ6-D
*TaTIFY11c-A*	TRIAE_CS42_4AS_TGACv1_307330_AA1019580				
*A0A1D5XCM3*	TIFYGG	198	TaJAZ7-A		
*TaTIFY11c-B*	TRIAE_CS42_4BL_TGACv1_320580_AA1043700				
*A0A1D5XHE2*	TIFYAG	261	TaJAZ7-B		
*TaTIFY11c-D*	Traes_4DL_7564D43A91		TIFFGG	210	TaJAZ7-D

IDs that begin with Traes were retrieved from phytozome, IDs that begin with TRIAE were retrieved from EnsemblPlant. When possible protein IDs from Uniprot were indicated.

**Fig 2:**
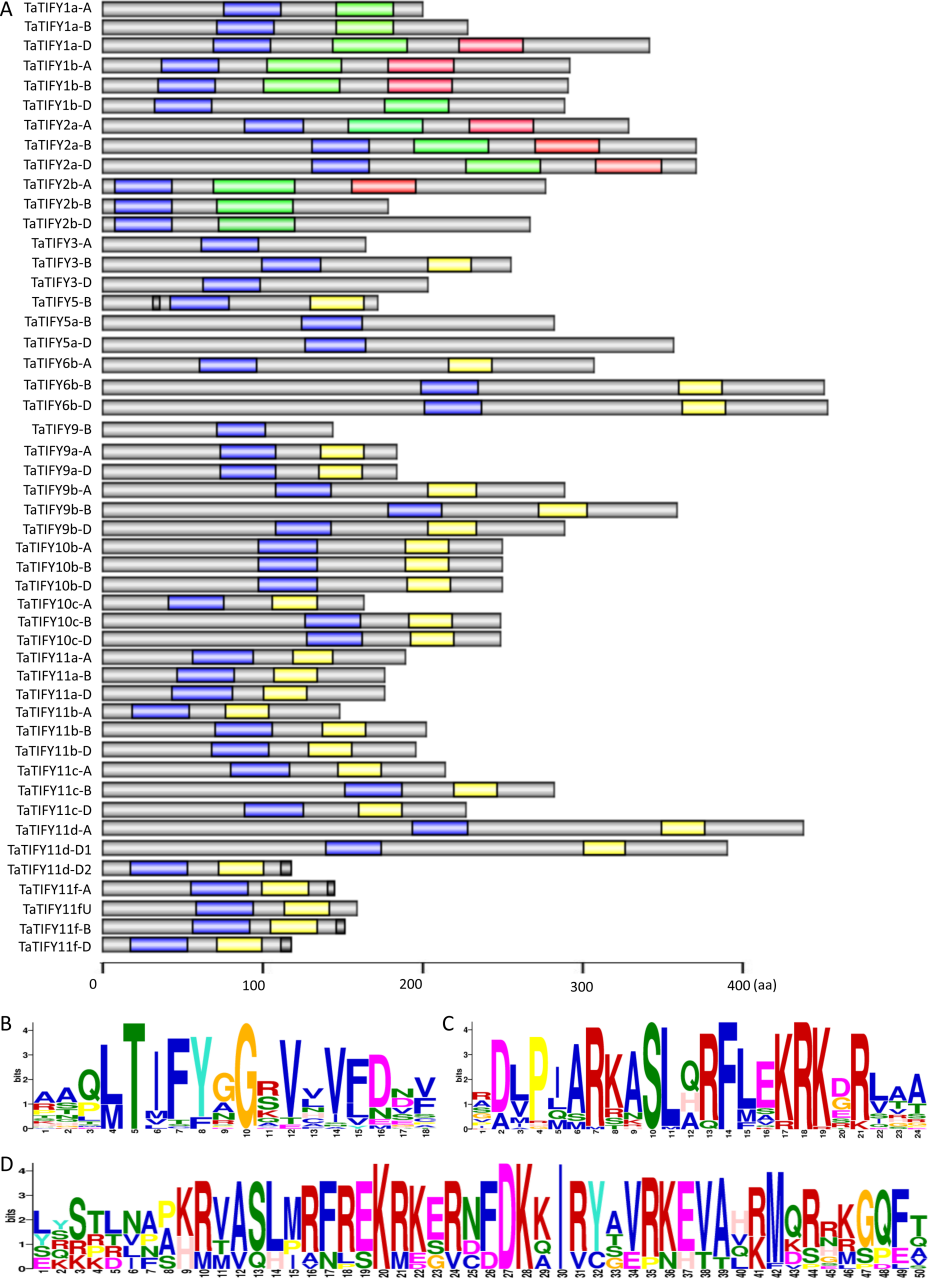
Conserved domains in wheat TIFY proteins. Schematic representation of 49 wheat TIFY proteins and conserved domains drawn with GPS tool (A). Blue boxes represent the TIFY domain, yellow highlight the Jas domain, green stand for divergent CCT motifs and red represent C2C2-GATA-Zinc-finger DNA binding domain. EAR motif is shown by black box. Grey bars represent non-conserved sections. The scale at the bottom (indicating the number of amino acid) corresponds to the proteins length. Consensus of sequences conservation of TIFY/ZIM domain (B), Jas motifs (C) and GATA domain (D) using MEME.

Phylogenetic analyses identified 4 groups within the 18 *Ta*TIFY proteins (Fig 1 and Table 1). The phylogenetic tree revealed that 4 major clades of TIFY proteins are present in the 3 monocots (wheat, rice and Brachypodium) (Figs 1 and 2A). Proteins in the TIFY3, TIFY5/6 and TIFY10/11 groups possess, in addition to the typical TIFY motif (Figs 2B and S1), the canonical Jas domain characteristic of the JAZ repressors (Figs 2C and S2). Proteins in group TIFY1/2 (*Ta*TIFY1a, 1b, 2a, 2b in the case of wheat) possess, besides the TIFY motif, a CCT domain and a C2C2-GATA-Zinc finger DNA binding domain, which are typical of ZIM-subfamily proteins (Figs 2D and S3). The PEAPOD domain is typical of the TIFY4 family in *Arabidopsis*[13,23] but no proteins showing similarity to *At*TIFY4 have been found in any of the studied monocot species (wheat, rice or *Brachypodium*). Finally, we did not find in *T*. *aestivum* any ortholog of TIFY8 as observed for *Brachypodium*[20,21].

Among *Arabidopsis* TIFY proteins, members of the *At*TIFY5 and *At*TIFY11 clades contain the “Ethylene-responsive element binding factor-associated amphiphilic repression” (EAR) domains able to directly recruit the repressor TPL independently on NINJA[18,40]. Five wheat proteins (*Ta*TIFY5B, *Ta*TIFY11f-A, *Ta*TIFY11f-B, *Ta*TIFY11f-D and *Ta*TIFY11d-D2) also contain a classical EAR motif (LxLxL) at their C-terminus (Figs 2A and S4). However, no *Ta*TIFYs show the NDLxxP EAR motif, occurring only in the AtTIFY5 proteins. EAR motifs are also present in the same clades of orthologous monocot TIFY members (*Bd*TIFY5, *Bd*TIFY11d, *Bd*TIFY11e, *Bd*TIFY11f and *Os*TIFY5 and *Os*TIFY11e)[20,21].

The analysis of the genomic localization of wheat TIFY genes showed that the 18 groups are located on chromosome 2 (n = 4), 4 (n = 5), 5 (n = 3), 6 (n = 2) and 7 (n = 4) and distributed along the three subgenomes. Only one gene, TaTIFY11f-U, could not be assigned to any specific chromosome (U). Recently, Wang et al.,[29] described 34 different common wheat *TaJAZ* genes grouped in 14 homeologous subsets, which possess Jas and TIFY domains. Within the 18 TIFY homeologous proteins identified here, the 14 homeologous JAZ have been retrieved and classified in three distinct groups (JAZ1, JAZ2 and JAZ3). However, the previously named *Ta*JAZ4, *Ta*JAZ5, *Ta*JAZ11 and *Ta*JAZ14 exhibit a TIFY domain but a divergent CCT domain, not a canonical Jas domain[29]. In addition, these *Ta*JAZ proteins retain a GATA domain and should therefore be classified as ZIM-like proteins rather than JAZ proteins (Fig 2)[13]. Within the TIFY3, TIFY5/6 and TIFY10/11 groups of wheat JAZ proteins, the Jas domain is highly conserved (Fig 2C), with conservation of the residues involved in COI1-JAZ interaction (L/VPXARR/K, Fig 2C), JAZ-JA interaction (Ala at position 6, Fig 2C) but also in MYC2 binding (RXXSLXRFLXXR, Fig 2C).

### Expression analyses of wheat *TIFY* genes under stress conditions

**Fig 3:**
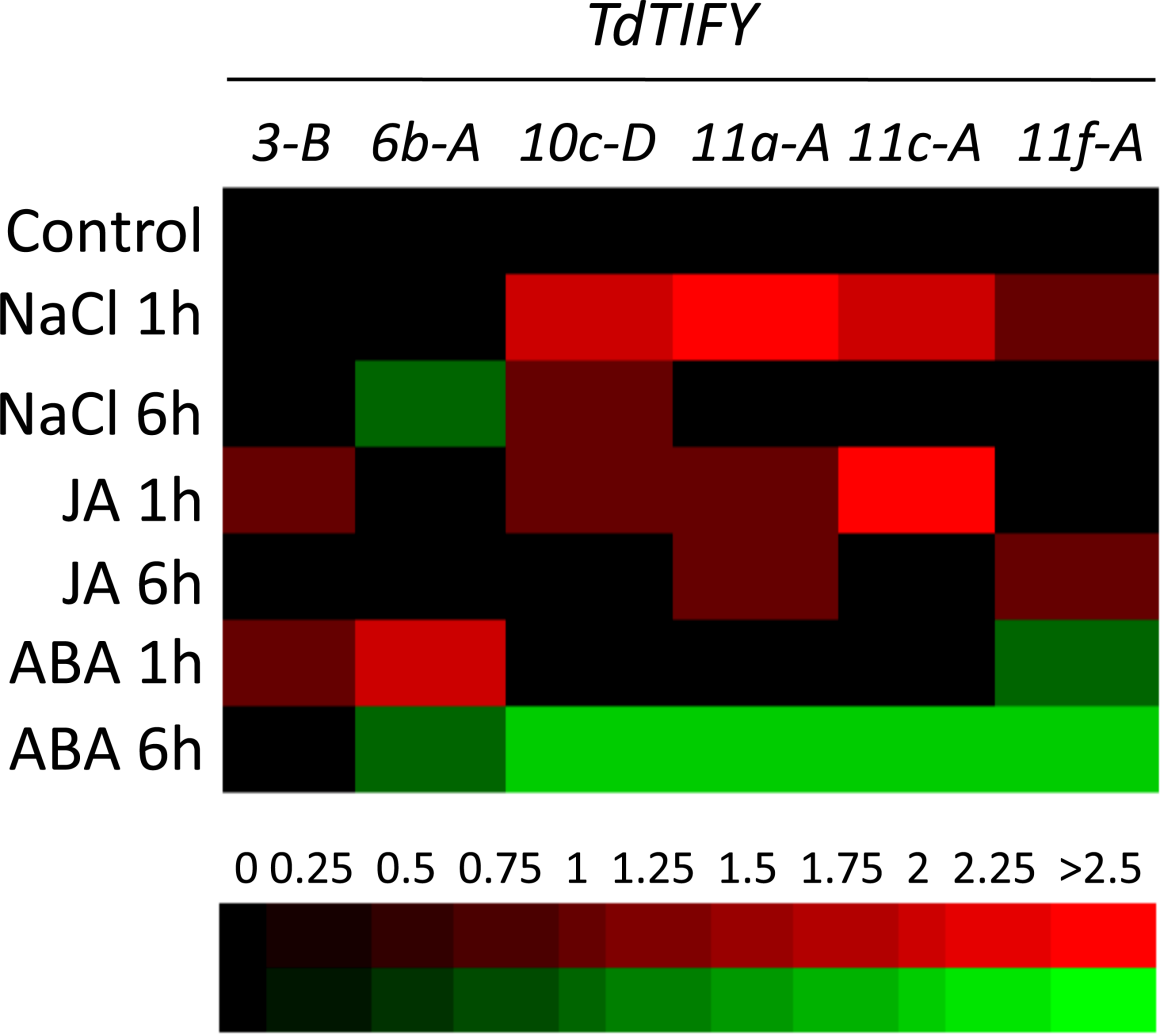
Expression pattern of 6 durum wheat *TIFY* genes in response to stresses. Seven-day-old durum wheat seedlings were treated with NaCl (150 mM), JA (50 μM) or ABA (100 μM) for 1 or 6 hours. Relative expression of *TIFY* genes was analyzed by quantitative real-time qPCR using wheat actin as control. Log2 transformed values were used to generate the color-coded heatmap. The color-coded scale bar is indicated below the heatmap.

Transcriptional regulation of *JAZ* genes in response to abiotic stresses has been reported in several plant species[19–21,28,29]. To assess the expression of durum wheat *JAZ/TIFY* genes under various stress treatments, we selected six wheat genes orthologs of monocot salt-induced *JAZ/TIFY* genes[20,21]. Expression analyses were performed by qRT-PCR on the well-characterized Oum Rabiaa Tunisian durum wheat variety after either 1 or 6 hours exposure to JA (50 μM), ABA (100 μM), or NaCl (150 mM). As shown in Fig 3, *TdTIFY10c*, *TdTIFY11a*, *TdTIFY11c* and *TdTIFY11f* were quickly induced by salt treatment. This induction is transient since 6 hours after salt treatment the basal level of *TdTIFY* expression is restored, with the exception of *TdTIFY10c*, which is still slightly induced (Fig 3). Among the tested genes, *TdTIFY11a* showed the strongest expression in response to salt. In contrast, salt stress did not alter *TdTIFY3* expression, whereas it slightly down-regulated *TdTIFY6*. JA treatment up-regulated all tested *JAZ/TIFY* genes except *TdTIFY6b* (Fig 3). ABA down-regulated the expression of most of the genes with the exception of *TdTIFY3* and *TdTIFY6b* (Fig 3).

In summary, this analysis reveals that *TdTIFY* genes are differentially regulated in response to salinity and hormone treatments.

### Identification and characterization of *TdTIFY11a*

*TdTIFY11a* is highly induced by salt, mildly up-regulated in response to JA and not induced by ABA. The *TdTIFY11a* ortholog Os*TIFY11a/OsJAZ9* exhibited a similar expression pattern and its overexpression conferred salt and drought stress tolerance in rice transgenic plants[20]. Therefore, we analyzed the putative role of *TdTIFY11a* in salt-stress responses. The *TdTIFY11a* gene from *Triticum durum Oum Rabiaa* variety was isolated. Its nucleotide sequence was 99, 93 and 91% identical to common wheat genes *TaTIFY11a-B*, *TaTIFY11a-A* and *TaTIFY11a-D*, respectively. The *TdTIFY11a* encoded protein is 100% identical to TaTIFY11a-B but only 80% to *Ta*TIFY11a-D and *Ta*TIFY11a-A (S5 Fig).

Next, two GFP-tagged *TdTIFY11a* constructs (the full-length sequence or a truncated version without the Jas domain) were expressed in *Arabidopsis* plants under the constitutive CaMV *35S* promoter. Two lines for each construct were chosen based on the highest *Td*TIFY11a-GFP protein accumulation (ie. lines 8 and 17 for the full-length version and lines 40 and 57 for the ΔJas construct) (S6 Fig).

**Fig 4:**
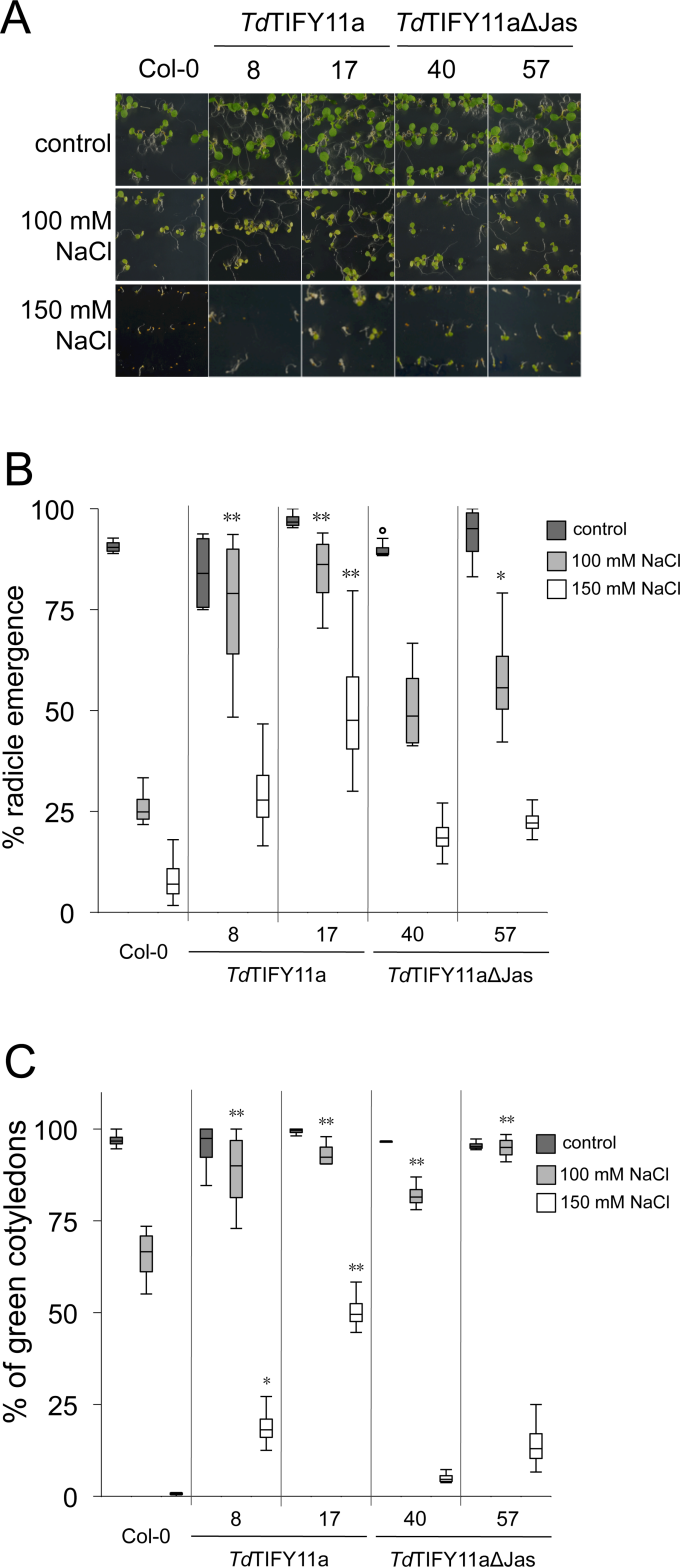
Over-expression of *TdTIFY11a* variants confers salt tolerance to *Arabidopsis* seedlings. (A) 7-day-old seedlings (N = 20 to 50) of the different over-expressing lines germinated on control media or in presence of 100 or 150 mM NaCl. Percentage of radicle emergence of 2 day-old seedlings (B), percentage of seedlings with green cotyledons at day 5 after germination (C) and on control media or supplemented with NaCl (100 or 150 mM). Data presented as box-plots; horizontal lines are medians, boxes show the interquartile range and error bars show the full data range. The experiments were repeated at least 4 times with similar results. Asterisks (B-C) indicate statistical significance (One-way ANOVA with post-hoc Tukey HSD, * p<0.05, ** p<0.01).

The phenotypes of these transgenic lines were compared to wild type (WT) under control and salinity conditions. Seeds were germinated in the presence of 100 and 150 mM NaCl concentrations. Germination rates were measured as radicle emergence 2 days after germination, whereas cotyledon greening was recorded 5 days after germination. Under control conditions, the full-length *Td*TIFY11a-GFP and *Td*TIFY11aΔJas-GFP lines germinated equivalently to WT control (Fig 4A–4B). However, in presence of salt all the *TIFY11a* over-expressing lines exhibited significantly higher germination rates compared to WT seeds (Fig 4A–4B). This enhanced salt tolerance is more pronounced on full-length *Td*TIFY11a-GFP than *Td*TIFY11aΔJas-GFP seedlings. For example, in the presence of 150 mM NaCl, both *Td*TIFY11a-GFP lines had 3–5 fold increases, while *Td*TIFY11aΔJas-GFP lines showed only 2 fold increase in radicle emergence compared to WT. The difference in radicle emerge of *Td*TIFY11a line 17 is significantly higher than that of wild-type seedlings (p-value <0.01; Fig 4B).

A significant (30%) increase in cotyledon greening was also observed at day 5 on the transgenic lines germinated on salt containing medium in comparison with wild-type control plants (Fig 4C). The *Td*TIFY11aΔJas-GFP line 17 showed the highest cotyledon greening ratio (50%) at 150 mM (Fig 4C). To confirm that *Td*TIFY11a-GFP and *Td*TIFY11aΔJas-GFP proteins still accumulate after few days of germination in presence of salt, the levels of the *Td*TIFY11a-GFP were monitored; the truncated *Td*TIFY11aΔJas-GFP is detected at similar levels in the absence or presence of salt stress treatment. In the case of full length *Td*TIFY11a-GFP, less protein seems to accumulate after germination in high salinity conditions (S6 Fig). Next, we reasoned that *TdTIFY11a* overexpression could influence the expression of endogenous *AtJAZ* levels. For this purpose, the expression in the four *AtJAZ* genes in *Td*TIFY11a transgenic lines was analyzed by qRT-PCR in control or salt stress conditions (see S7 Fig). In basal conditions, levels of *AtJAZs* in OE-TdTIFY11a transgenic lines are very similar to those in wild-type seedlings (S7 Fig). In response to salt treatment, the expression of many *AtJAZ* genes was induced in wild-type seedlings, and this salt-induction was generally higher in OE-TdTIFY11a transgenic lines (S7 Fig). In contrast to seedlings, adult plants of *Td*TIFY11a-GFP lines exposed to increasing salt concentrations or drought stress failed to show significant difference in tolerance (S8 Fig). Therefore the increase in salt stress tolerance conferred by *Td*TIFY11a overexpression is limited to early stage development of *Arabidopsis* plants.

Finally, the responses of plants overexpressing *Td*TIFY11a variants to JA were analyzed. The phenotypes of the *Td*TIFY11a and *Td*TIFY11aΔJas-GFP lines were compared to wild type under control or exogenous JA treatment. JA induced a similar root growth inhibition and anthocyanin accumulation in wild-type and all *Td*TIFY11a transgenic lines (S9 Fig) indicating that the overexpression of the wheat TIFY gene in *Arabidopsis* did not alter its JA responses.

Altogether, the results show that the over-expression of full-length and truncated *TdTIFY11a* confers higher germination rates under high salinity conditions.

## Discussion

Plant adaptation to their changing environment is orchestrated by complex regulatory networks where JA-Ile plays a primary role in regulating defense mechanisms and abiotic stress responses[2,4,28]. JA-Ile acts through a well-described signaling pathway, in which JAZ proteins are central negative regulators of JA responses[7,9]. The JAZ family belongs to the larger TIFY super-family, well characterized in eudicots such as *Arabidopsis thaliana* but still poorly known in wheat. To date, 14 homeologous *JAZ* loci have been identified in the common wheat (*Triticum aestivum* L.) and their expression patterns characterized in response to stress treatments[29]. However, the identification of the complete TIFY super-family in wheat was lacking. Our study identifies 49 TIFY proteins encoded by 49 genes located in the three different wheat subgenomes. The identification in wheat of all orthologous proteins of rice and *Brachypodium* indicates exhaustiveness of our analysis. Phylogenetic and domain analyses show that *Ta*JAZ4, *Ta*JAZ5, *Ta*JAZ11 and *Ta*JAZ14[29] contain a CCT motif and GATA motif typical of ZIM-like proteins; therefore *Ta*JAZ4, *Ta*JAZ5, *Ta*JAZ11 and *Ta*JAZ14 are not “bona-fide” JAZ proteins and should be best referred as *Ta*TIFY1a, *Ta*TIFY1b, *Ta*TIFY2a and *Ta*TIFY2b respectively (Fig 1).

No orthologous protein of AtTIFY8 could be identified in wheat and *Brachypodium*. Likewise no TIFY7 could be identified in *Brachypodium*, rice and wheat, indicating that these classes of proteins might be specific of eudicots but absent in monocots. Five wheat TIFY proteins contain a canonical EAR motif (LxLxL) (Figs 2 and S4), supporting the hypothesis of a direct recruitment of TPL to negatively regulate JA-mediated transcription and the conservation in wheat of the JAZ-TPL repression mechanism[40]. Wheat orthologous of PPD proteins were not identified, in agreement with their absence in rice and *Brachypodium*, supporting the hypothesis that the PPD subfamily is only present in dicots[41].

The Jas motif of the wheat JAZ is highly conserved (Figs 2C and S2), including the specific residues directly mediating COI1-JAZ complex formation, hormone binding and JAZ interaction with MYC TFs[6,12]. This high conservation of the functional residues within the Jas motif suggests that wheat JAZ proteins are able to interact with the corresponding key wheat JA-pathway components in a similar fashion as described in *Arabidopsis*.

The expression pattern of six *TdTIFY* genes showed that they are differentially regulated by JA, ABA and salt (Fig 3). Interestingly, their regulation is comparable to that of the rice and *Brachypodium* orthologous *TIFY* genes. For instance, the three monocot *TIFY11a* orthologous genes are all up-regulated by salt, slightly induced by JA but not affected by ABA[20,21] (this work). Hence, their expression might be mediated by conserved regulatory mechanisms.

The expression of *OsTIFY11a/OsJAZ9* under drought-inducible promoter confers drought and salt stress tolerance to rice plants, without altering the responses to JA[20,21]. Likewise, over-expressing two durum wheat ortholog *TdTIFY11a* variants in *Arabidopsis* does not alter responses to JA but increases germination efficiency under salt stress conditions, including higher radicle emergence rates and enhanced seedling establishment (Figs 4C, S8 and S9). These are important agronomic traits in the context of abiotic stress tolerance—*ie*. seeds are able to germinate despite adverse conditions. Both transgenic lines over-expressing either full-length *Td*TIFY11a or the truncated *Td*TIFY11aΔJas, are similarly salt stress tolerant, suggesting that the Jas domain may not be critical for the positive regulatory role of TIFY11a in salt stress tolerance. However, *Td*TIFY11aΔJas does retain the ZIM/TIFY domain mediating the interaction of several *At*JAZ proteins with AtWRKY57, whose over-expression confers salt tolerance in *Arabidopsis* plants[42,43]. Similar to the case of *Td*TIFY11a over-expression plants, the *At*WRKY57-mediated stress tolerance only occurs in seed germination and early post-germination growth, whereas adult plants fail to show salt tolerance. This suggests that the role of *Td*TIFY11a in salt tolerance may rely on the activity of the wheat WRKY57 orthologs. Besides, salt and drought tolerance conferred by *OsTIFY11a*/*OsJAZ9* over-expression was reported only in young rice seedlings[20], similarly to the case of *Td*TIFY11a over-expression plants. Several *Os*JAZ proteins directly interact with *Os*bHLH148, which in turn modulates the expression of JA-regulated ion transporters and promotes stress tolerance[31,44]. In addition, *Os*TIFY11a/*Os*JAZ9 also interacts with and regulates *Os*bHLH062, a TF that directly binds to the promoters of the ion transporter genes such as OsHAK21 to regulate salt tolerance in rice plants[31]. It is therefore reasonable that *Td*TIFY11a may act in a similar manner in *Arabidopsis*, conferring salt stress tolerance via the *Os*bHLH148 and/or *Os*bHLH062 orthologous-signaling pathway. However, the truncated *Td*TIFY11aΔJas lacking the Jas motif would not directly interact with these bHLH TFs. It is feasible that the *Td*TIFY11aΔJas variant would dimerize with additional JAZ proteins and consequently indirectly interfere with these or other TFs. Future identification and characterization of the orthologous wheat bHLH148 and/or *Os*bHLH062 orthologous will test this hypothesis.

On another hand, heterologous expression of *TdTIFY11a* constructs may interfere with the endogenous expression of *AtJAZ* genes, which in turn could confer germination tolerance in high salinity conditions. In basal conditions, the endogenous levels of *JAZs* in *Td*TIFY11a and *Td*TIFY11aΔJas transgenic lines are very similar to those in wild-type seedlings, providing evidence against the hypothesis that altered basal *JAZ* expression may prime germination tolerance (S7 Fig). As previously reported, most *JAZ* genes are induced in response to high salinity stress. This salt-induction of *JAZ* genes is generally higher in *Td*TIFY11a transgenic lines; therefore, it is plausible that this enhanced *JAZ* expression may depend on the ectopic *TdTIFY11a* over-expression. However, the enhanced *JAZ* expression in the *Td*TIFY11a transgenic lines is not very high, approximately twice that of wild-type plants (S7 Fig). Therefore, the hypothesis that variation in *JAZ* expression may affect the salt tolerance response in OE-TdTIFY11a transgenic lines requires further studies.

The rice RSS3 protein forms a ternary complex with *Os*bHLH094 and *Os*TIFY11a/*Os*JAZ9[45]. *Os*RSS3 and *Os*TIFY11a synergistically regulate the expression of JA-induced salt-responsive genes[45]; therefore the enhanced salt tolerance of *Td*TIFY11a over-expressing plants may also involve the orthologous *RSS3* wheat gene. Finally, *Os*TIFY11a/*Os*JAZ9 also interact with additional TFs involved in tolerance to stresses other than drought; for example, *Os*TIFY11a directly interacts with and represses *Os*MYB30, a key TF regulating cold tolerance in rice[46]. Thus, it is reasonable that *Td*TIFY11a may regulate additional, still unidentified wheat TFs to mediate salt stress tolerance.

Why the over-expression of TdTIFY11a exhibits enhanced salt tolerance only at early stages of plant development (*ie*. seedling establishment) is unclear. The quicker turnover of *Td*TIFY proteins in mature tissues compare to early stage seedlings may account for the lack of stress tolerance in adult plants. Alternatively, specific spatiotemporal expression (*ie*. only expressed at seedling stage) of different TFs regulated by *Td*TIFY11a may explain the developmental specificity.

Plants growth under high salt stress conditions show partial decreases of full-length TdTIFY11a but not of *Td*TIFY11aΔJas protein level (S6 Fig). In this context, salt stress induces accumulation of JA-Ile in plants[47–49]. Therefore, the differential protein stability between *Td*TIFY11a and *Td*TIFY11aΔJas may depend on the salt-induced accumulation of JA-Ile that in turn triggers full-length *Td*TIFY11a degradation. In contrast, the stability of TdTIFY11aΔJas (lacking the Jas motif mediating JA-Ile dependent COI1 interaction) is not affected by salt stress.

In conclusion, we identified 49 typical *TIFY* genes, grouped into 16 homeologous loci, in common wheat divided into two subfamilies, namely *ZML* and *JAZ*. Over-expression of *Td*TIFY11a in *Arabidopsis* conferred higher germination rates under high salinity conditions indicating a relevant role of JAZ proteins in abiotic stress responses.

## Supporting information

S1 Table Primer pairs used for the QRT-PCR. (PDF)

S1 Fig Multiple sequence alignment of the conserved TIFY domain of several wheat TIFY proteins. Alignment of the 49 *Triticum aestivum* TIFY proteins showing the conserved TIFY domain. Protein IDs indicated are the same as listed in Table 1. The alignment was performed with MEGA6.06 using CLUSTALW and the BLOSUM matrix.
(PDF)

S2 Fig Multiple sequence alignment of the canonical Jas motif of several wheat TIFY proteins. The alignment of the sequences of the conserved Jas motif of canonical wheat JAZ proteins (of the TIFY3, TIFY5/6 and TIFY10/11 clades) were employed (A). The alignment was performed with MEGA6.06 using the BLOSUM matrix. (B) Sequence logo of the Jas motif using MEME on the same proteins aligned in A.
(PDF)

S3 Fig Multiple sequence alignment of the CCT motif and GATA domain of several wheat TIFY proteins. The alignment of the sequences of the conserved CCT motif (A) and GATA domain (C) of wheat TIFY proteins belonging to the group TIFY1/2 were employed. The sequence logo for the CCT motif (B) and GATA domain (D) were generated with MEME.
(PDF)

S4 Fig Multiple sequence alignment of the EAR motif in wheat TIFY proteins. The alignment of the sequences of the conserved EAR motif (A) of five wheat TIFY proteins belonging to the group TIFY5 and TIFY11 were employed. (B) The sequence logo for the EAR motif.
(PDF)

S5 Fig Alignment of *Ta*TIFY11a and *Td*TIFY11a sequences. A) Multiple protein alignment of *Ta*TIFY11a-A, -B, -D and *Td*TIFY11aperformed with MEGA6 (MUSCLE matrix). Residues highlighted in blue are conserved among all proteins whereas residues in red are conserved between *Ta*TIFY11a-B and *Td*TIFY11a. B) Phylogenetic tree performed with MEGA6 based on the multiple alignment in A using Neighbour-joining method with BLOSUM matrix and 1000 bootstrap iterations. C) Multiple cDNA alignment performed with MEGA6 (MUSCLE matrix). Nucleotides highlighted in blue are conserved among all genes. Nucleotides marked in red are conserved between *TaTIFY11a-B* and *TdTIFY11a*, whereas the only two divergent nucleotides between *TaTIFY11a-B* and *TdTIFY11a* are highlighted in yellow.
(PDF)

S6 Fig Protein accumulation of TdTIFY11a-GFP variants. Immunoblot analyses of *Td*TIFY11a-GFP and actin protein levels in *35S*:*Td*TIFY11a-GFP (full length, line 8 and 17), *Td*TIFY11aΔJas-GFP (line 57); wild-type Col-0 (WT) was included as a negative control. Seeds were germinated in control media (-) or in presence of 100 mM NaCl (+) and seven-day-old seedlings were used for the analysis. Protein molecular weights are indicated at the sides.
(PDF)

S7 Fig *JAZ* gene expression in *Td*TIFY11a transgenic lines. Gene expression analysis of *JAZ* genes in 5-day-old Arabidopsis seedlings treated with mock solution or 150 mM NaCl. Relative expression of *JAZ* genes was analyzed by quantitative real-time qPCR using actin 8 as housekeeping control. Each biological sample consisted of tissue pooled from 10–15 plants. Data show mean ± SD of three to four technical replicates.
(PDF)

S8 Fig Abiotic stress responses of adult *Td*TIFY11aΔJas-GFP plants. 3-week-old plants wild-type and *Td*TIFY11aΔJas-GFP (line 57) were exposed to increasing salt concentrations (100 to 400 mM NaCl) or drought stress. Wild-type and *Td*TIFY11aΔJas-GFP showed similar responses to these abiotic stresses.
(PDF)

S9 Fig Analyses of over-expression of *Td*TIFY11a variants in response to JA treatment. Wild-type (Col-0) and transgenic *Td*TIFY11a seedlings (N = 10 to 30) were germinated in absence (control) or presence of 50 μM JA. Nine days after germination, root growth (A) (mm) and anthocyanin accumulation (B) [Abs(530nm)/fresh weight (mg)] were measured. *coi1-1* seedlings were included as control. Data presented as box-plots; horizontal lines are medians, boxes show the interquartile range and error bars show the full data range. The experiments were repeated at least 2 times with similar results. Letters stand for statistical differences (One-way ANOVA with post-hoc Tukey HSD, p<0.01).
(PDF)
