# Supplementary material for: Characterization of wheat (Triticum aestivum) TIFY family and role of Triticum Durum TdTIFY11a in salt stress tolerance
Source: PLoS One. 2018 Jul 18;13(7):e0200566. doi: 10.1371/journal.pone.0200566 (PMC6051620; doi:10.1371/journal.pone.0200566)
Supplement: S1 Table — (PDF) [file pone.0200566.s001.pdf]

Supplementary Table 1: Quantitative RT-PCR primers used in this study

Primer name	Sequences
TIFY11aF	5'-ATGCCGCCGATGGCGACCA-3'
TIFY11aR	5'-CGTCTTGCCCTTCTTCTTTG-3'
TIFY11fF	5'-CCTCATGCCCGGATCCGAAGT-3'
TIFY11fR	5'-AGAGGGTAAGCGTGAGACGA-3'
TIFY6bF	5'- ATCTTCAGGCCAGTTGGTTG-3'
TIFY6bF	5'-TCTCTGTGCACGATGAGGAC-3'
TIFY11cF	5'- GCCAGTACATGAGGGAGCAG-3'
TIFY11cR	5'- ATGAGCTCCCTGGCCTTCT-3'
TIFY3F	5'- ACCAGAGAAGGCTCAAGCAA-3'
TIFY3R	5'- GCTGAAGGGAGTGTCTCCTG-3'
TIFY10cF	5'- GCCACAACTGACAACCAAGA-3'
TIFY10cR	5'- CGCCGATGAGCTGTTAATTT-3'
ActF	5'-GTGCCCATTTACGAAGGATA -3'
ActR	5'- GAAGACTCCATGCCGATCAT -3'
