# Supplementary material for: Characterization of wheat (Triticum aestivum) TIFY family and role of Triticum Durum TdTIFY11a in salt stress tolerance
Source: PLoS One. 2018 Jul 18;13(7):e0200566. doi: 10.1371/journal.pone.0200566 (PMC6051620; doi:10.1371/journal.pone.0200566)
Supplement: S1 Fig — Alignment of the 49 Triticum aestivum TIFY proteins showing the conserved TIFY domain. Protein IDs indicated are the same as listed in Table 1. The alignment was performed with MEGA6.06 using CLUSTALW and the BLOSUM matrix. (PDF) [file pone.0200566.s002.pdf]

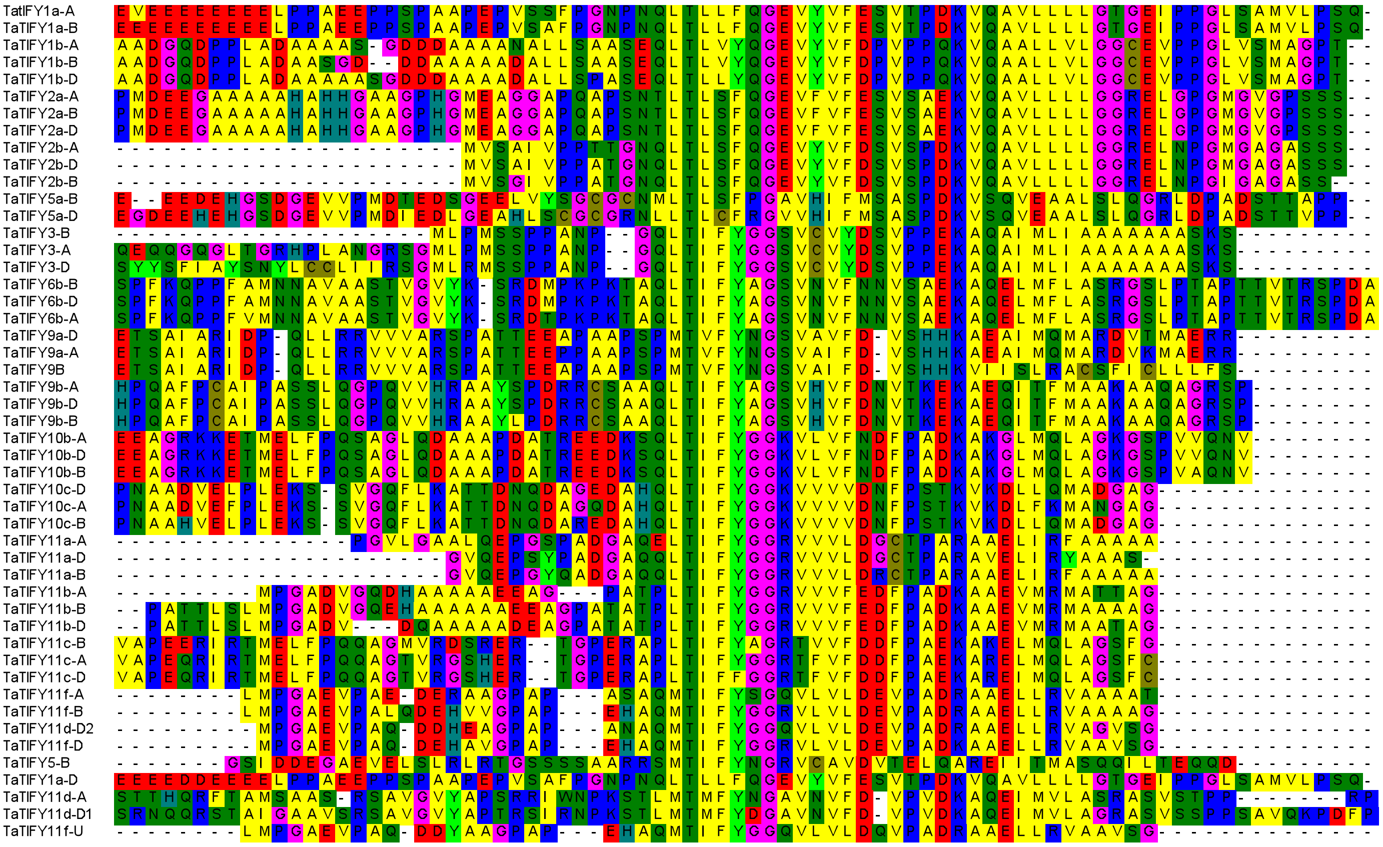

Supplementary Figure S1. Multiple sequence alignment of the conserved TIFY domain of several wheat TIFY proteins.

Alignment of the 49 Triticum aestivum TIFY proteins showing the conserved TIFY domain. Protein IDs indicated are the same as listed in Table 1. The alignment was performed with MEGA6.06 using CLUSTALW and the BLOSUM matrix.
