# Supplementary material for: Characterization of wheat (Triticum aestivum) TIFY family and role of Triticum Durum TdTIFY11a in salt stress tolerance
Source: PLoS One. 2018 Jul 18;13(7):e0200566. doi: 10.1371/journal.pone.0200566 (PMC6051620; doi:10.1371/journal.pone.0200566)
Supplement: S2 Fig — The alignment of the sequences of the conserved Jas motif of canonical wheat JAZ proteins (of the TIFY3, TIFY5/6 and TIFY10/11 clades) were employed (A). The alignment was performed with MEGA6.06 using the BLOSUM matrix. (B) Sequence logo of the Jas motif using MEME on the same proteins aligned in A. (PDF) [file pone.0200566.s003.pdf]

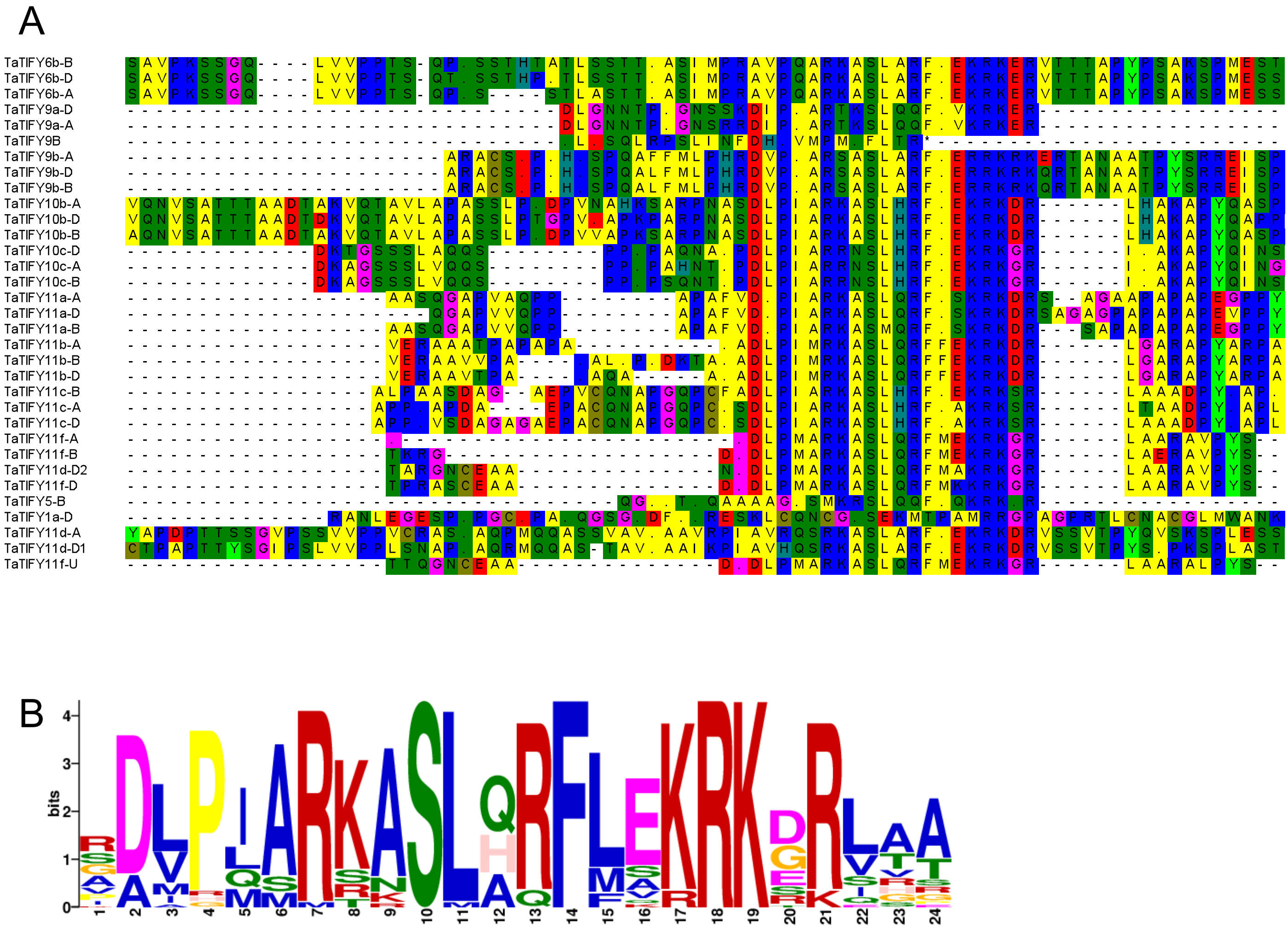

Supplementary Figure S2. Multiple sequence alignment of the canonical Jas motif of several wheat TIFY proteins.

The alignment of the sequences of the conserved Jas motif of canonical wheat JAZ proteins (of the TIFY3, TIFY5/6 and TIFY10/11 clades) were employed (A). The alignment was performed with MEGA6.06 using the BLOSUM matrix. (B) Sequence logo of the Jas motif using MEME on the same proteins aligned in A.
