# Supplementary material for: Characterization of wheat (Triticum aestivum) TIFY family and role of Triticum Durum TdTIFY11a in salt stress tolerance
Source: PLoS One. 2018 Jul 18;13(7):e0200566. doi: 10.1371/journal.pone.0200566 (PMC6051620; doi:10.1371/journal.pone.0200566)
Supplement: S3 Fig — The alignment of the sequences of the conserved CCT motif (A) and GATA domain (C) of wheat TIFY proteins belonging to the group TIFY1/2 were employed. The sequence logo for the CCT motif (B) and GATA domain (D) were generated with MEME. (PDF) [file pone.0200566.s004.pdf]

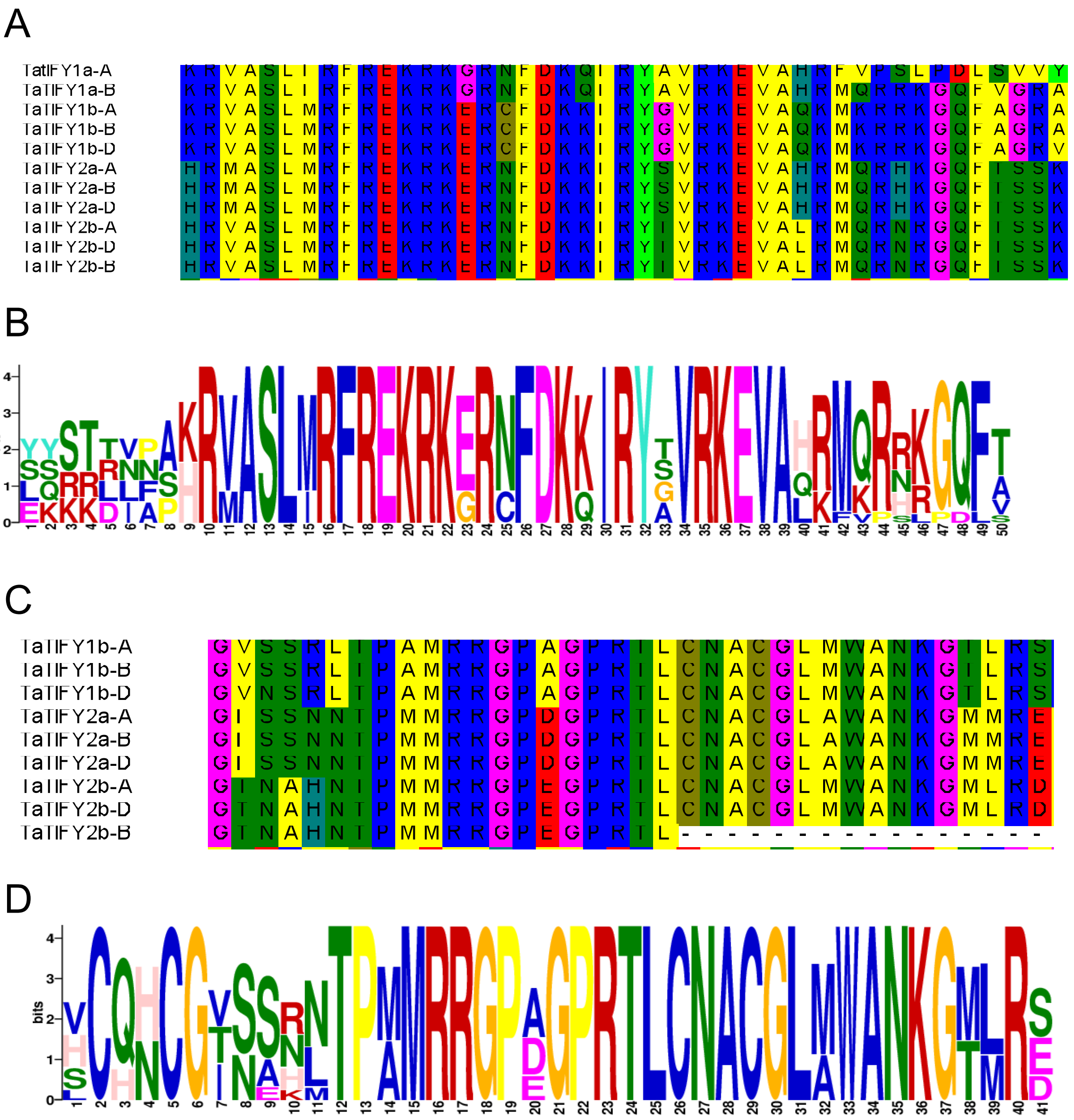

Supplementary Figure S3. Multiple sequence alignment of the CCT motif and GATA domain of several wheat TIFY proteins.

The alignment of the sequences of the conserved CCT motif (A) and GATA domain (C) of wheat TIFY proteins belonging to the group TIFY1/2 were employed. The sequence logo for the CCT motif (B) and GATA domain (D) were generated with MEME.
