# Supplementary material for: Characterization of wheat (Triticum aestivum) TIFY family and role of Triticum Durum TdTIFY11a in salt stress tolerance
Source: PLoS One. 2018 Jul 18;13(7):e0200566. doi: 10.1371/journal.pone.0200566 (PMC6051620; doi:10.1371/journal.pone.0200566)
Supplement: S4 Fig — The alignment of the sequences of the conserved EAR motif (A) of five wheat TIFY proteins belonging to the group TIFY5 and TIFY11 were employed. (B) The sequence logo for the EAR motif. (PDF) [file pone.0200566.s005.pdf]

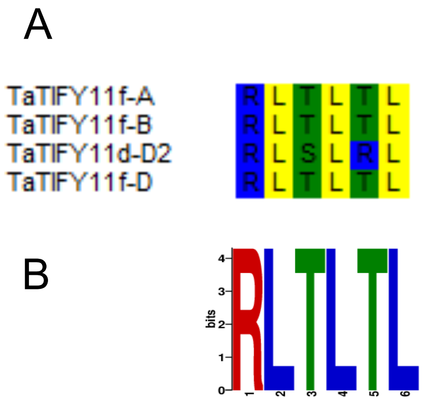

Supplementary Figure S4. Multiple sequence alignment of the EAR motif in wheat TIFY proteins.

The alignment of the sequences of the conserved EAR motif (A) of wheat five TIFY proteins belonging to the group TIFY5 and TIFY11 were employed. The sequence logo for the EAR motif (B).
