# Supplementary material for: Characterization of wheat (Triticum aestivum) TIFY family and role of Triticum Durum TdTIFY11a in salt stress tolerance
Source: PLoS One. 2018 Jul 18;13(7):e0200566. doi: 10.1371/journal.pone.0200566 (PMC6051620; doi:10.1371/journal.pone.0200566)
Supplement: S5 Fig — A) Multiple protein alignment of TaTIFY11a-A, -B, -D and TdTIFY11aperformed with MEGA6 (MUSCLE matrix). Residues highlighted in blue are conserved among all proteins whereas residues in red are conserved between TaTIFY11a-B and TdTIFY11a. B) Phylogenetic tree performed with MEGA6 based on the multiple alignment in A using Neighbour-joining method with BLOSUM matrix and 1000 bootstrap iterations. C) Multiple cDNA alignment performed with MEGA6 (MUSCLE matrix). Nucleotides highlighted in blue are conserved among all genes. Nucleotides marked in red are conserved between TaTIFY11a-B and TdTIFY11a, whereas the only two divergent nucleotides between TaTIFY11a-B and TdTIFY11a are highlighted in yellow. (PDF) [file pone.0200566.s006.pdf]

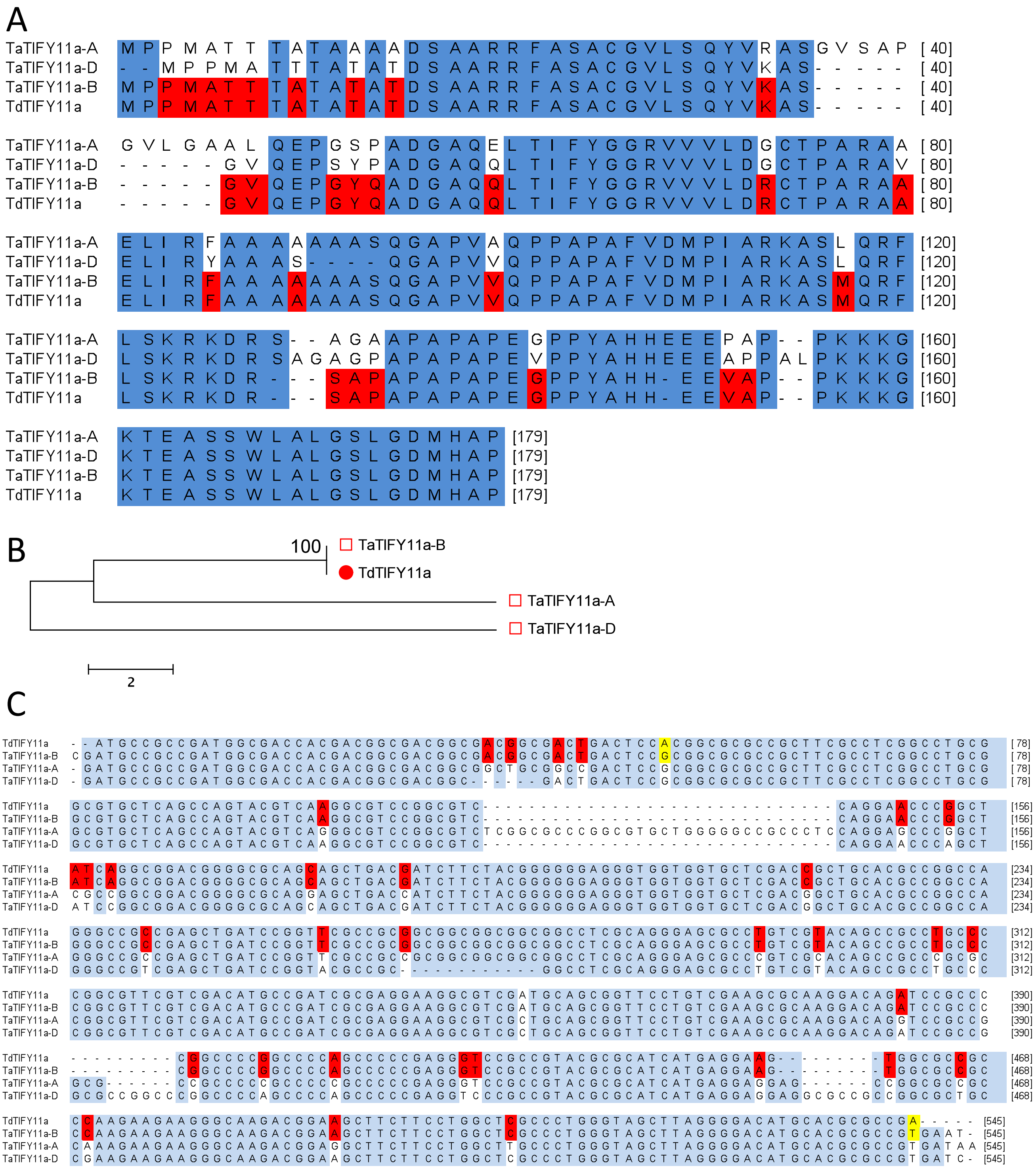

Supplementary Figure S5. Alignment of *Ta*TIFY11a and *Td*TIFY11a sequences.

A) Multiple protein alignment of *Ta*TIFY11a-A, -B, -D and *Td*TIFY11aperformed with MEGA6 (MUSCLE matrix). Residues highlighted in blue are conserved among all proteins whereas residues in red are conserved between *Ta*TIFY11a-B and *Td*TIFY11a.

B) Phylogenetic tree performed with MEGA6 based on the multiple alignment in A using Neighbour-joining method with BLOSUM matrix and 1000 bootstrap iterations.

C) Multiple cDNA alignment performed with MEGA6 (MUSCLE matrix). Nucleotides highlighted in blue are conserved among all genes. Nucleotides marked in red are conserved between *TaTIFY11a-B* and *TdTIFY11a*, whereas the only two divergent nucleotides between *TaTIFY11a-B* and *TdTIFY11a* are highlighted in yellow.
