# Supplementary material for: Characterization of wheat (Triticum aestivum) TIFY family and role of Triticum Durum TdTIFY11a in salt stress tolerance
Source: PLoS One. 2018 Jul 18;13(7):e0200566. doi: 10.1371/journal.pone.0200566 (PMC6051620; doi:10.1371/journal.pone.0200566)
Supplement: S6 Fig — Immunoblot analyses of TdTIFY11a-GFP and actin protein levels in 35S:TdTIFY11a-GFP (full length, line 8 and 17), TdTIFY11aΔJas-GFP (line 57); wild-type Col-0 (WT) was included as a negative control. Seeds were germinated in control media (-) or in presence of 100 mM NaCl (+) and seven-day-old seedlings were used for the analysis. Protein molecular weights are indicated at the sides. (PDF) [file pone.0200566.s007.pdf]

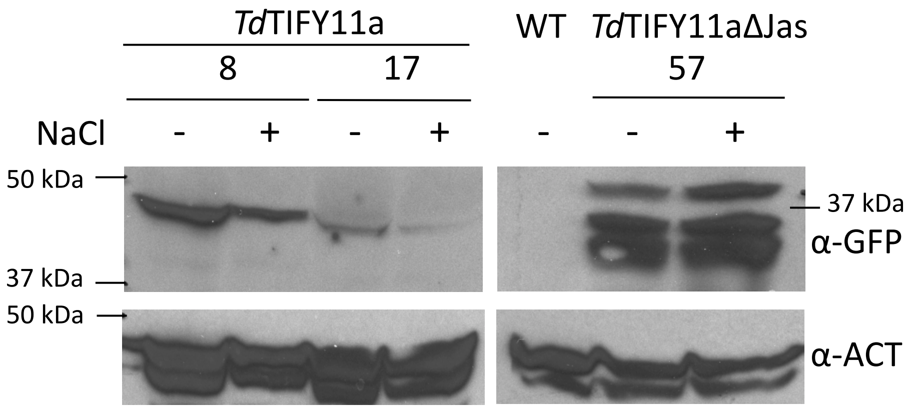

Supplementary Figure S6. Protein accumulation of *Td*TIFY11a-GFP variants.

Immunoblot analyses of *Td*TIFY11a-GFP and actin protein levels in 35S:TdTIFY11a-GFP (full length, line 8 and 17), *Td*TIFY11aΔJas-GFP (line 57); wild-type Col-0 (WT) was included as a negative control. Seeds were germinated in control media (-) or in presence of 100 mM NaCl (+) and seven-day-old seedlings were used for the analysis. Protein molecular weights are indicated at the sides.
