# Supplementary material for: Characterization of wheat (Triticum aestivum) TIFY family and role of Triticum Durum TdTIFY11a in salt stress tolerance
Source: PLoS One. 2018 Jul 18;13(7):e0200566. doi: 10.1371/journal.pone.0200566 (PMC6051620; doi:10.1371/journal.pone.0200566)
Supplement: S7 Fig — Gene expression analysis of JAZ genes in 5-day-old Arabidopsis seedlings treated with mock solution or 150 mM NaCl. Relative expression of JAZ genes was analyzed by quantitative real-time qPCR using actin 8 as housekeeping control. Each biological sample consisted of tissue pooled from 10–15 plants. Data show mean ± SD of three to four technical replicates. (PDF) [file pone.0200566.s008.pdf]

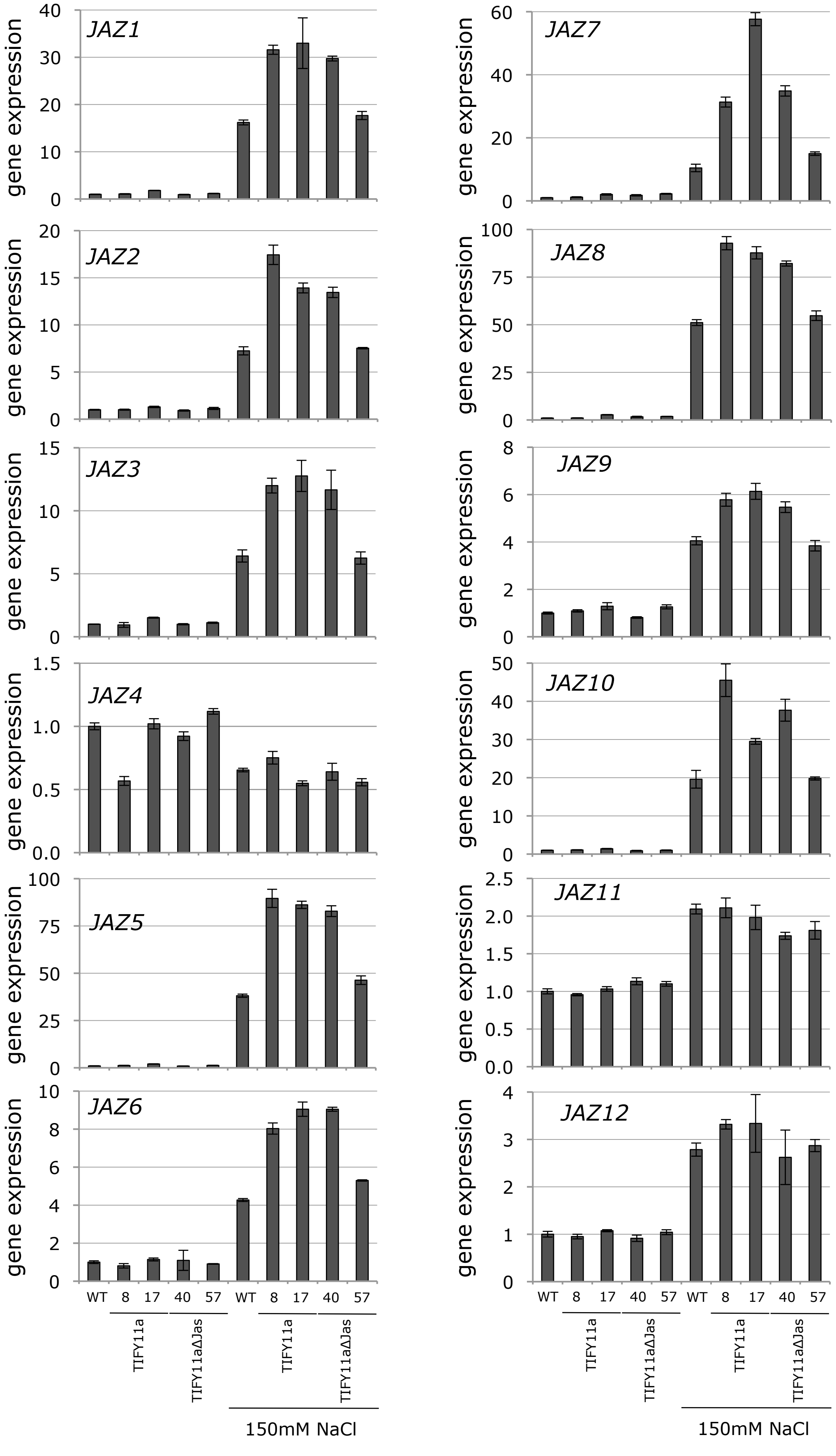

JAZ1
JAZ2
JAZ3
JAZ4
JAZ5
JAZ6
JAZ7
JAZ8
JAZ9
JAZ10
JAZ11
JAZ12
gene expression
WT
8
17
40
57
TIFY11a
TIFY11aΔJas
150mM NaCl

Supplementary Figure S7. *JAZ gene* expression in *Td*TIFY11a transgenic lines.

Gene expression analysis of *JAZ* genes in 5-day-old Arabidopsis seedlings treated with mock solution or 150 mM NaCl. Relative expression of *JAZ* genes was analyzed by quantitative real-time qPCR using actin 8 as housekeeping control. Each biological sample consisted of tissue pooled from 10-15 plants. Data show mean ± SD of three to four technical replicates.
