# Supplementary material for: Characterization of wheat (Triticum aestivum) TIFY family and role of Triticum Durum TdTIFY11a in salt stress tolerance
Source: PLoS One. 2018 Jul 18;13(7):e0200566. doi: 10.1371/journal.pone.0200566 (PMC6051620; doi:10.1371/journal.pone.0200566)
Supplement: S8 Fig — 3-week-old plants wild-type and TdTIFY11aΔJas-GFP (line 57) were exposed to increasing salt concentrations (100 to 400 mM NaCl) or drought stress. Wild-type and TdTIFY11aΔJas-GFP showed similar responses to these abiotic stresses. (PDF) [file pone.0200566.s009.pdf]

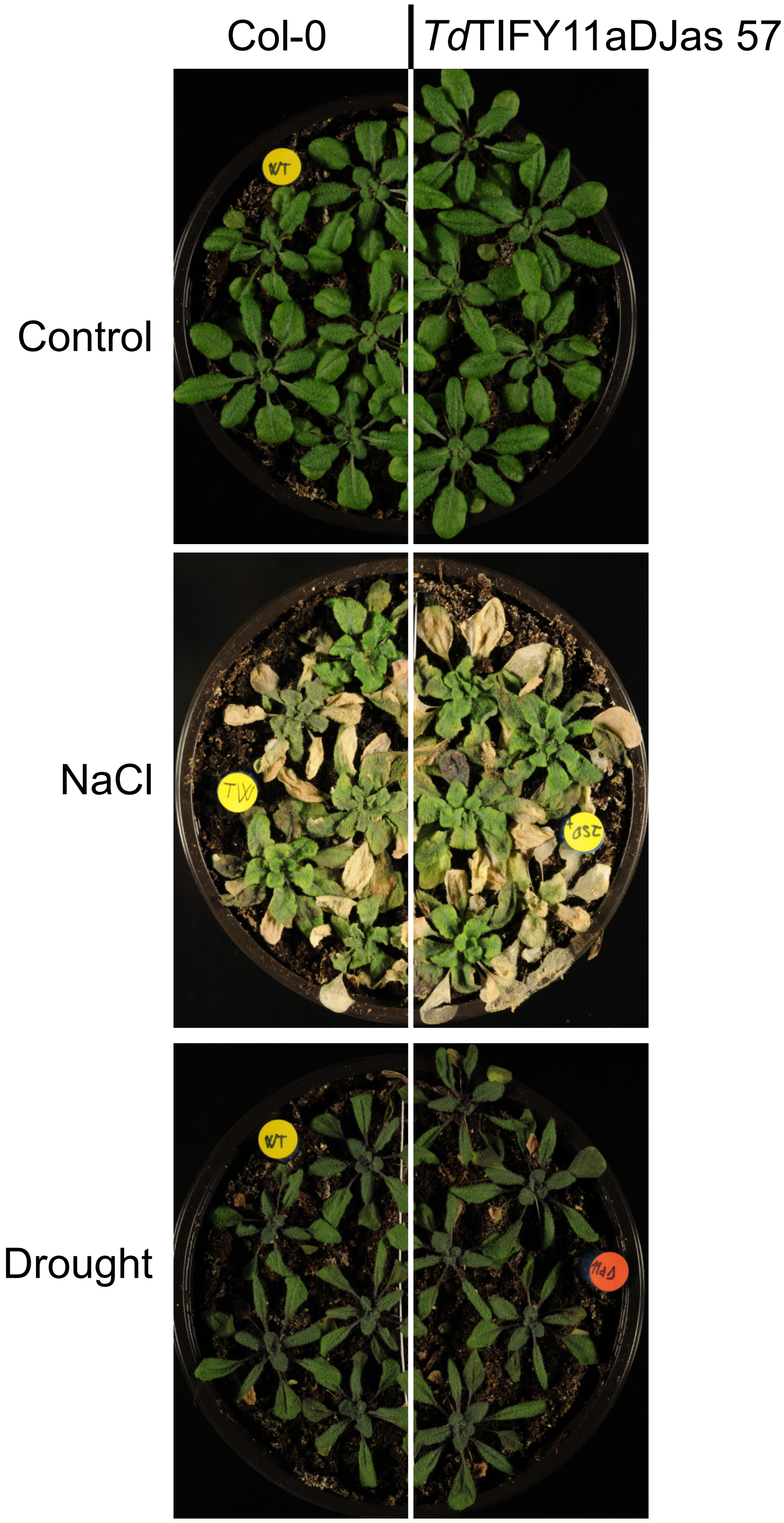

Supplementary Figure S8. Abiotic stress responses of adult *Td*TIFY11aΔJas-GFP plants. 3-week-old plants wild-type and *Td*TIFY11aΔJas-GFP (line 57) were exposed to increasing salt concentrations (100 to 400 mM NaCl) or drought stress. Wild-type and *Td*TIFY11aΔJas-GFP showed similar responses to these abiotic stresses.
