# Supplementary material for: Characterization of wheat (Triticum aestivum) TIFY family and role of Triticum Durum TdTIFY11a in salt stress tolerance
Source: PLoS One. 2018 Jul 18;13(7):e0200566. doi: 10.1371/journal.pone.0200566 (PMC6051620; doi:10.1371/journal.pone.0200566)
Supplement: S9 Fig — Wild-type (Col-0) and transgenic TdTIFY11a seedlings (N = 10 to 30) were germinated in absence (control) or presence of 50 μM JA. Nine days after germination, root growth (A) (mm) and anthocyanin accumulation (B) [Abs(530nm)/fresh weight (mg)] were measured. coi1-1 seedlings were included as control. Data presented as box-plots; horizontal lines are medians, boxes show the interquartile range and error bars show the full data range. The experiments were repeated at least 2 times with similar results. Letters stand for statistical differences (One-way ANOVA with post-hoc Tukey HSD, p<0.01). (PDF) [file pone.0200566.s010.pdf]

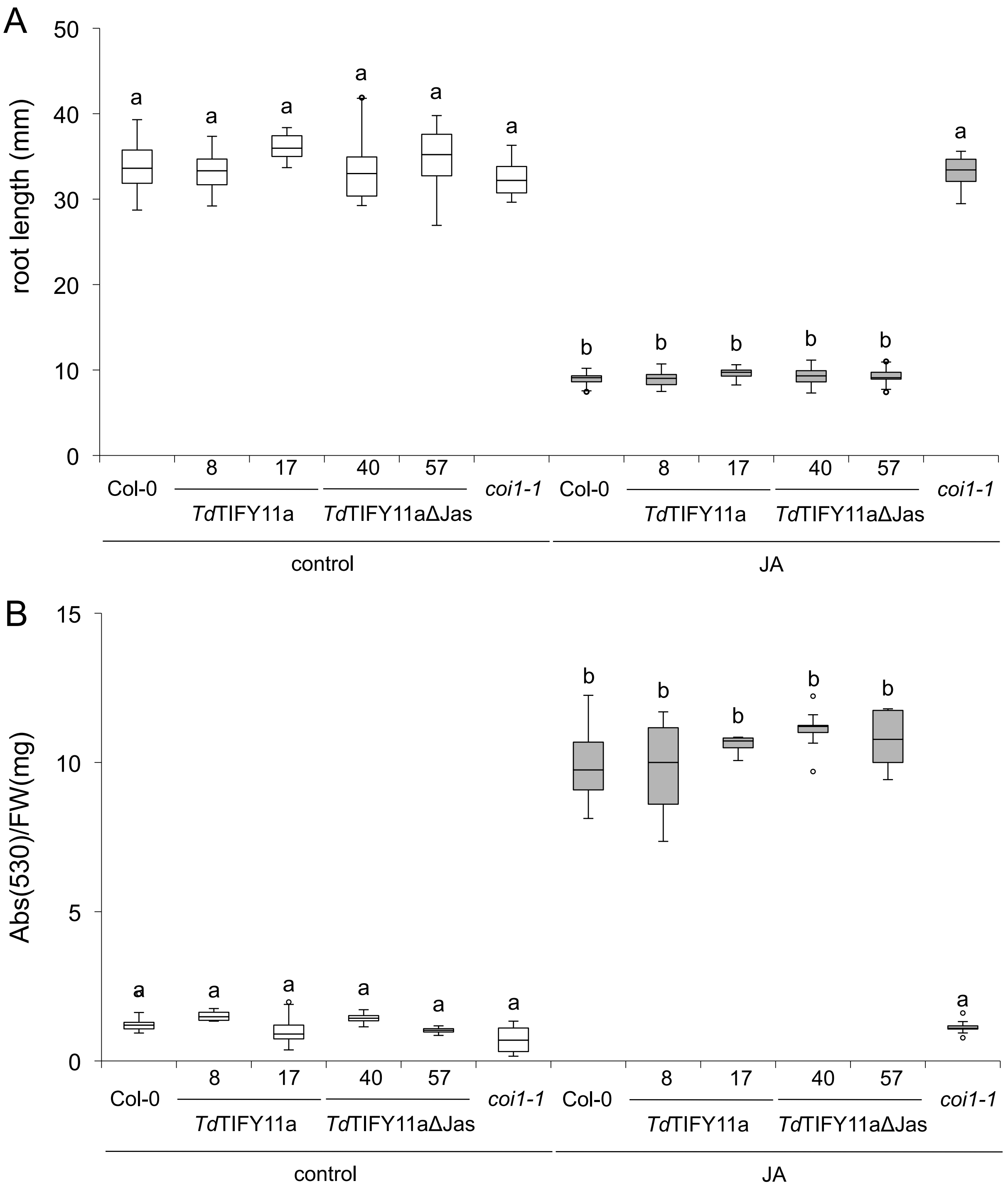

Supplementary Figure S9. Analyses of over-expression of *Td*TIFY11a variants in response to JA treatment.

Wild-type (Col-0) and transgenic *Td*TIFY11a seedlings were germinated in absence (control) or presence of 50 mM JA. Nine days after germination, root growth (A) (mm) and anthocyanin accumulation (B) [Abs(530nm)/fresh weight (mg)] were measured. coi1-1 seedlings were included as control. The experiments were repeated at least 2 times with similar results. Letters stand for statistical differences (One-way ANOVA with post-hoc Tukey HSD, $p<0.01$).
